# Depression of lncRNA MINCR antagonizes LPS-evoked acute injury and inflammatory response via miR-146b-5p and the TRAF6-NFkB signaling

**DOI:** 10.1186/s10020-021-00367-3

**Published:** 2021-10-03

**Authors:** Wei Gao, Ying Zhang

**Affiliations:** 1 Department of Critical Care Medicine, The Second Hospital of Shandong University, Jinan, 250033 Shandong People’s Republic of China; 2 Department of Respiratory, The Second Hospital of Shandong University, No.247 Beiyuan Avenue, Jinan, 250033 Shandong People’s Republic of China

**Keywords:** MINCR, miR-146b-5p, TRAF6/NF-κB, LPS, ALI/ARDS

## Abstract

**Background:**

Inflammation plays an important role in the development of acute lung injury (ALI) and acute respiratory distress syndrome (ARDS). The long non-coding RNA (lncRNA) MINCR is closely related to inflammation injury. This study was performed to explore the protective effects and mechanisms of MINCR in lipopolysaccharide (LPS)-induced lung injury and inflammation.

**Methods:**

The expression levels of MINCR and miR-146b-5p in lung tissue status were detected by using quantitative real-time polymerase chain reaction (qRT-PCR), hematoxylin and eosin staining, immunohistochemical staining, and terminal deoxynucleotidyl transferase dUTP nick end labeling assay. Enzyme-linked immunosorbent assay and Western blotting analysis were used to detect the expression of inflammatory factors such as tumor necrosis factor (TNF)-α, interleukin (IL)-6, and IL-10 in lung tissue. The relationship between MINCR, miR-146b-5p, and TRAF6 was explored using bioinformatics analysis and luciferase assay.

**Results:**

The expression levels of MINCR were increased in a mouse model of LPS-induced ALI and small airway epithelial cells (SAECs). shMINCR resulted in increased cell viability and decreased apoptosis, which protected against LPS-induced cell damage. shMINCR can inhibit the formation of neutrophil extracellular traps, neutrophil numbers, myeloperoxidase activity, and the production of inflammatory cytokines IL-6, IL-1β, and TNF-α induced by LPS. The silencing of miR-146b-5p reversed the effects of MINCR on LPS-induced lung damage. Sh-MINCR decreased the expression levels of TRAF6 and p-P65 in LPS-induced SAECs and lung tissues. Co-transfection of sh-MINCR with miR-146b-5p inhibitor reversed the effect of sh-MINCR on the expression of TRAF6 and p-P65.

**Conclusions:**

MINCR may induce alveolar epithelial cell injury and inflammation and aggravate the progression of ALI/ARDS through miR-146b-5p and TRAF6/NF-κB pathways, which would provide a promising target for the treatment of ALI/ARDS.

## Background

Acute lung injury (ALI) is a disease that is widely distributed worldwide. It is an acute progressive insufficiency or respiratory failure caused by various internal and external factors of the lung, with a mortality rate of up to 40% (Tao et al. 2019). The common pathological manifestations of ALI are damage to alveolar epithelial cells, an increase in pulmonary capillary permeability, thickening of the alveolar wall, and the release of inflammatory factors in the alveoli cavity (Ochroch et al. 2019). With reduced lung volume, reduced lung compliance can develop into acute respiratory distress syndrome (ARDS) in later stages (Cao et al. 2018). The pathogenesis of ARDS is affected by coagulation and fibrinolysis. The function of the human coagulation system is enhanced, whereas the fibrinolysis system is inhibited, leading to a large area of thrombosis and a large amount of fibrin deposition, resulting in vascular blockage and microcirculation structure damage (Bender et al. 2020). The lipopolysaccharide (LPS)-induced lung injury model has been widely used in studies on lung injury (Zhu et al. 2013). There are no specific response methods for ALI. Currently, auxiliary ventilation and drug therapy are the main clinical response methods (Chiumello et al. 2019). Therefore, it is important to elucidate the regulatory mechanisms and signaling pathways involved in the pathological process of ALI to provide targeted drugs for clinical treatment.

At present, the pathogenesis of ALI is considered to be an inflammatory response of the body. Infection is a common risk factor for ALI, and LPS is the main endotoxin (Cheung et al. 2018). Long non-coding RNAs (lncRNAs) play key roles in regulating inflammatory immune genes (Wu et al. 2018). These can act as regulatory factors and exert their functions by interacting with proteins, RNAs, and DNA to regulate their expression at transcriptional and post-transcriptional levels (Liu et al. 2009). Several studies have investigated the regulation of lncRNAs on epigenetics and gene transcription and their involvement in disease occurrence and development, particularly in Alzheimer’s disease, cancer, and other diseases (Wang et al. 2017; Xie et al. 2019). lncRNAs also play roles in controlling the balance of inflammatory responses (Fava et al. 2017). For example, lncRNA CASC2 reduces apoptosis of pulmonary epithelial cells and improves ALI by regulating the miR-144-3p/AQP1 axis (Li et al. 2018a). A better understanding of lncRNAs will provide new ideas for exploring the nature of sepsis and regulating the uncontrolled inflammatory cascade in sepsis. The lncRNA MINCR is a newly identified lncRNA that is abnormally expressed in many diseases (Hu et al. 2018). For example, the expression levels of MINCR are significantly increased in primary liver cancer tissues (Lyu et al. 2019). However, the functions and mechanisms of MINCR in ALI have not yet been studied.

MicroRNAs (miRNAs) are involved in many important pathophysiological processes. In recent years, studies have explored their regulation in tumors, inflammation, and other aspects in molecular biology, cell biology, and clinical medicine (Emma et al. 2020). miRNAs are widely involved in the immune regulation of systemic inflammatory response syndrome and ALI (Iacona et al. 2018). In the process of the systemic inflammatory response, miRNAs regulate the expression of various inflammatory genes and transcription factors, the proliferation of various inflammatory cells, the production and release of various cytokines and inflammatory mediators, and the expression of various inflammatory factor receptors (Sedgeman and Michell 2019). Studies have found that miR-155, miR-17-92, and miR-223 participate in the regulation of ALI caused by toxicosis (Feng et al. 2017). MiR-146b-5p plays different roles in the progression of different diseases (Sheng et al. 2018). For example, it can inhibit the proliferation of osteosarcoma cells (Zhu et al. 1136). However, the expression and function of miR-146b-5p in ALI remain unclear. NF-κB is a crucial factor in cell proliferation and differentiation (Jian et al. 2019). It acts on the rank receptor on the cell membrane and transmits the signal to TRAF6, which is an important link molecule in cell formation and can cause the chain reaction of downstream signaling and promote its proliferation (Kanaya et al. 2018). Isoprolactone has been reported to inhibit TRAF6 ubiquitination and reduce ALI development (Ding et al. 2019). Therefore, it was speculated that MINCR regulated the TRAF6-NF-kB axis through miR-146b-5p to regulate LPS-induced ALI. This study explored the mechanism of action of MINCR in ALI.

## Materials and methods

### Ethics statement

All animal experiments were performed in accordance with the Guidelines for the Care and Use of Laboratory Animals (revised in 1996, publication No. 85-23) published by the National Institutes of Health, Nursing committee approval.

### Animal model of sepsis-induced ALI

Male C57BL/6 mice (N = 10, 6–8 weeks old, 20–24 g) were obtained from Wild River Laboratory Animal Technology Co., Ltd. (Beijing, China). The mice were randomly fed food and water. They were treated with 5 mg/kg LPS (*Escherichia coli* O111:B4 dissolved in saline, Sigma, St. Louis, MO, USA) by intratracheal instillation to induce ALI. The control group mice were anesthetized and sacrificed 24, 48, and 72 h after LPS administration. The mice were intraperitoneally injected with 30 mg/kg pentobarbital (WS20060401, Sinopharm Chemical, China). Bronchoalveolar lavage fluid (BALF), plasma, and tissue samples were collected by lavaging the lung with 1 mL PBS two times, and the red blood cells were lysed using lysis buffer followed by centrifugation. The trachea was intubated and the thorax was opened through a midline incision. The BALF was centrifuged, and the supernatant without cells was stored at − 80 °C for enzyme-linked immunosorbent assay (ELISA) as previously described (Gregoire et al. 2017, 2018).

### Lung histology and lung injury score

Lung tissues were collected from the mice 48 h after LPS instillation. The lungs were inflated with 10% buffered formalin, fixed with 10% buffered formalin, embedded in paraffin, and cut into 5-μm sections. The tissue sections were stained with hematoxylin and eosin (H&E), evaluated, and scored by a pathologist blinded to the experimental groups. To evaluate lung injury, seven independent random lung fields were evaluated per mouse for neutrophils in alveolar spaces, neutrophils in the interstitial spaces, hyaline membranes, proteinaceous debris filling the airspaces, and alveolar septal thickening, and weighted according to the relevance ascribed by the official American Thoracic Society workshop report on features and measurements of experimental acute lung injury in animals (Sheng et al. 2018). The resulting injury score was a continuous value between 0 and 1.

### Histological evaluation of lung injury

The lower right lung lobe was cut into thin sections (5 mm). The number of alveolar sacs was determined after H&E staining by investigators blinded to the experimental groups. Three lung sections from each mouse were examined. Three high-power fields (X100) were randomly selected in each section for observation. The average number of alveolar sacs per mouse was determined as the sum of all fields divided by 9. The crowded area was defined as thickened septa in the lung parenchyma where alveolar sacs were partly or completely collapsed under H&E-stained sections. A scoring system was used to quantify the crowded area by investigators blinded to the experimental groups, defined as follows: 0, no crowded area; 1, 15% of the area crowded.

High-power field was a crowded area; 2, 15–25% of the area crowded; 3, 25–50% of the area crowded; 4, 50–75% of the area crowded; 5, 75–100% of the area crowded.

Caspase-3 (Beyotime, Shanghai, China) activities were measured using colorimetric assay kits following the manufacturer’s instructions.

### Adenovirus gene delivery

Recombinant adenovirus containing mouse MINCR shRNA (Ad-MINCR-shRNA) and Ad-GFP were obtained from Obio Company (Shanghai Obio Technology Co., Ltd.). Seven days before ALI induction, adenovirus (1 × 10^9^ transducing units [TU], 50 μg/0.2 mL) was intravenously injected into mice. Control adenovirus (Ad-GFP) was injected into the control group. MiR-146b-5p antagomir, miR-146b-5p mimic, and NC were purchased from GenePharma (Shanghai, China). Thereafter, 50 μg of miR-146b-5p antagomir, miR-146b-5p mimic, or its negative control, was dissolved in 200 μL of sterile double-distilled water, and 50 μL of glucose solution was added to it. The tail vein of the mice was injected with 200 μL working solution (Chen et al. 2018; D’Alessio et al. 2016).

### Cell culture and LPS exposure

The human lung epithelial cell line small airway epithelial cells (SAECs) were obtained from Procell (Wuhan, China). SAECs were cultured in human collagen type IV-coated flasks (Sigma, St. Louis, MO, USA) supplemented with SAGM Bullet kit culture medium (Lonza, Allendale, NJ, USA). The cells were stored in DMEM supplemented with 10% fetal bovine serum (FBS). Adenovirus (1 × 10^9^ TU, 100 nmol/L) was used to infect the cells. The cells were cultured in media containing different doses of LPS.

### Cell viability assay

Cells were seeded into a 96-well plate at a density of 5000 cells/well. Thereafter, 100 μL of CCK8 solution (Liji, Shanghai, China) was added. After 4 h, the absorbance at 450 nm was measured using a microplate reader (Bio Tek Instruments, USA).

### Apoptosis assay

Cells were plated into 6-well plates at a density of 5 × 10^5^ cells/well and counted. The cells were centrifuged and 195 μL of Annexin V-FITC binding solution was added to them. After mixing with 5 μL of Annexin V-FITC and 10 μL propidium iodide staining solution, the cells were incubated for 10–20 min.

### Myeloperoxidase (MPO) activity analysis

The lung tissues preserved at − 80 °C were removed, liquid nitrogen was added to them, and they were rapidly grounded to powder in a mortar. Thereafter, 300 μL of RIPA lysate containing PMSF inhibitor was added. The sample was then placed on an ice plate for cracking for 10 min, and the lysate was collected after the sample was fully cracked. The collected lysate was centrifuged at 4 °C at 12,000 rpm, and the collected supernatant was carefully sucked. The concentrations of all samples were adjusted using RIPA lysate and detected using an MPO detection kit.

### ELISA

The expression levels of interleukin (IL)-6, tumor necrosis factor (TNF)-α, and IL-10 were determined using a kit (Fisher Scientific, Waltham, MA, USA). A Luminex 100/200 photometer (Luminex, Austin, Texas, USA) was used for cytokine quantification.

### Polymorphonuclear leukocyte (PMN) isolation and culture

BALF neutrophils were isolated by centrifugation. PMNs were collected and purified. Neutrophils were cultured in RPMI 1640 medium (Weike, Shanghai, China) containing 10% FBS.

### Western blotting analysis

Transfected cells were collected, and total proteins were extracted. Protein concentrations were determined using a bicinchoninic acid protein assay kit. Thereafter, proteins were separated using 10% polyacrylamide gel electrophoresis and blocked with 5% BSA for 1 h. After transferring onto PVDF membrane, the membrane was incubated with anti-TRAF6 (1:1000, Amyjet, Wuhan, China), anti-p65 (1:1000, Amyjet, Wuhan, China), anti-p-p65 (1:1000, Amyjet, Wuhan, China), anti-IL-10 (1:1000, Amyjet, Wuhan, China), anti-IL-6 (1:1000, Amyjet, Wuhan, China), anti-TNF-α (1:1000, Amyjet, Wuhan, China), and anti-GAPDH (1:1000, Amyjet, Wuhan, China) overnight, followed by incubation with anti-rabbit secondary antibody (1:1000, Amyjet, Wuhan, China) for 1 h (Sanaa and Seada 2020).

### Dual luciferase report assay

The wild type (WT) 3′-UTR of MINCR cDNA was synthesized by polymerase chain reaction (PCR) and cloned into pMIR-REPORT luciferase to generate the WT-MINCR 3′-UTR. MINCR 3′- was generated based on the WT-MINCR 3′-UTR A mutant of UTR and named as the resulting vector MUT-MINCR 3′-UTR. These vectors (pMIR-REPORT plasmid, WT-MINCR 3′-UTR, or MUT-MINCR 3′-UTR) and miR-146b-5p Mock or miR NC were transiently transfected into cells using Lipofectamine 3000 reagent (Invitrogen, Carlsbad, CA, USA). After 48 h of transfection, luciferase activity was measured using Promega (Madison, WI, USA). The putative binding site of TRAF6 to miR-146b-5p was also detected using the same method.

### RNA pull-down assay

Cells were transfected with biotin-labeled Bio-miR-146b-5p or Bio-miR-146b-5p-probes and Bio-NC-probes for 48 h and then collected. Cells were lysed using a complete RIP lysis buffer. Cell extracts were incubated with magnetic beads conjugated with anti Ago2 or anti-IgG antibodies. RNA was then purified by qRT (quantitative reverse transcription)-PCR analysis.

### qRT-PCR analysis

Total RNA was extracted using TRIzol reagent (Biosntech, Beijing, China). RNA quality was analyzed using a NanoDrop 1000 (Thermo Fisher Scientific, Inc.). SYBR Green (Takara Biotechnology, Co., Ltd., Dalian, China) was used for qRT-PCR. Amplification was performed using an ABI 7500 real-time PCR system. A qScript miRNA cDNA synthesis kit (Quantabio, Beverly, CA, USA) was used for cDNA synthesis. Gene expression levels were analyzed using the 2^−△△^CT method. The internal controls for the expression levels of miRNAs and lncRNAs were U6 and GAPDH, respectively. The primer sequences used were as follows.

- LncRNA MINCR, 5′-CTAATTCACCTGGCCCGAG-3′ (forward)
  5′-CGGCTAGAATCCCAAGG-3′ (reverse);
- miR-146b-5p, 5′-CTGAATGTGAAGAGGATGT-3′ (forward)
  5′-GTTCTTCGGCCTCCGGGCCC-3′ (reverse);
- miR negative control (miR-NC), 5′-UUCUCCGAACGUGUCACGUTT-3′ (forward)
  5′-UGAGAACUGAAUUCCAUAGGC UG-3′ (reverse);
- IL-1β, 5′-CTTCAGGCAGCCAGTATCAATC-3′ (forward)
  5′-TGCAGTGGTCTTATGGGAACGT-3′ (reverse);
- MCP1, 5′-ACAACCAAGGCCTTCCTTAC-3′ (forward)
  5′-TCTCATTCCCACGATTCCCCAG-3′ (reverse);
- TNF-α, 5′-CGAGTGGCAAGCCTGGAGGCC-3′ (forward)
  5′-GTCTTGGAGATCCATCCGGTTG-3′ (reverse);
- GAPDH, 5′-AACGAACCTTTCATTGAC-3′ (forward)
  5′-CACCACTCTTACAGCAACT-3′ (reverse).

### Immunohistochemical staining

The Power Vision two-step method was used and the images were analyzed using Image-Pro Plus 6.0 image color analysis software. Each slice was randomly selected from five fields of view (×200 or ×400), and the expression of F4/80 and the presence of neutrophils and macrophages were evaluated by immunohistochemistry.

### Terminal deoxynucleotidyl transferase biotin-dUTP nick end labeling (TUNEL) assay

Cells from different treatment groups were collected, smeared, washed, and fixed with PBS. After reaction on ice with 100 μL of permeabilization buffer for 2‒5 min, the cells were washed with PBS. Thereafter, 50 μL of the pre-cooled reaction solution was added to the slide and incubated for 60–90 min. Cell staining was observed using a fluorescence microscope. Positive staining with nuclear DNA fragmentation was detected by fluorescence microscopy and quantified by the number of TUNEL-positive cells in ten fields per section and at least five sections per lung (at ×200 magnification).

### Statistical methods

Data are represented as the mean ± standard deviation and analyzed using SPSS19.0. Differences among multiple groups were analyzed using one-way ANOVA. The least significant difference test was used for subsequent analyses. *P* < 0.05 indicating that the difference was significant.

## Results

### MINCR was upregulated in ALI mouse model and LPS-injured alveolar epithelial cells

**Fig. 1:**
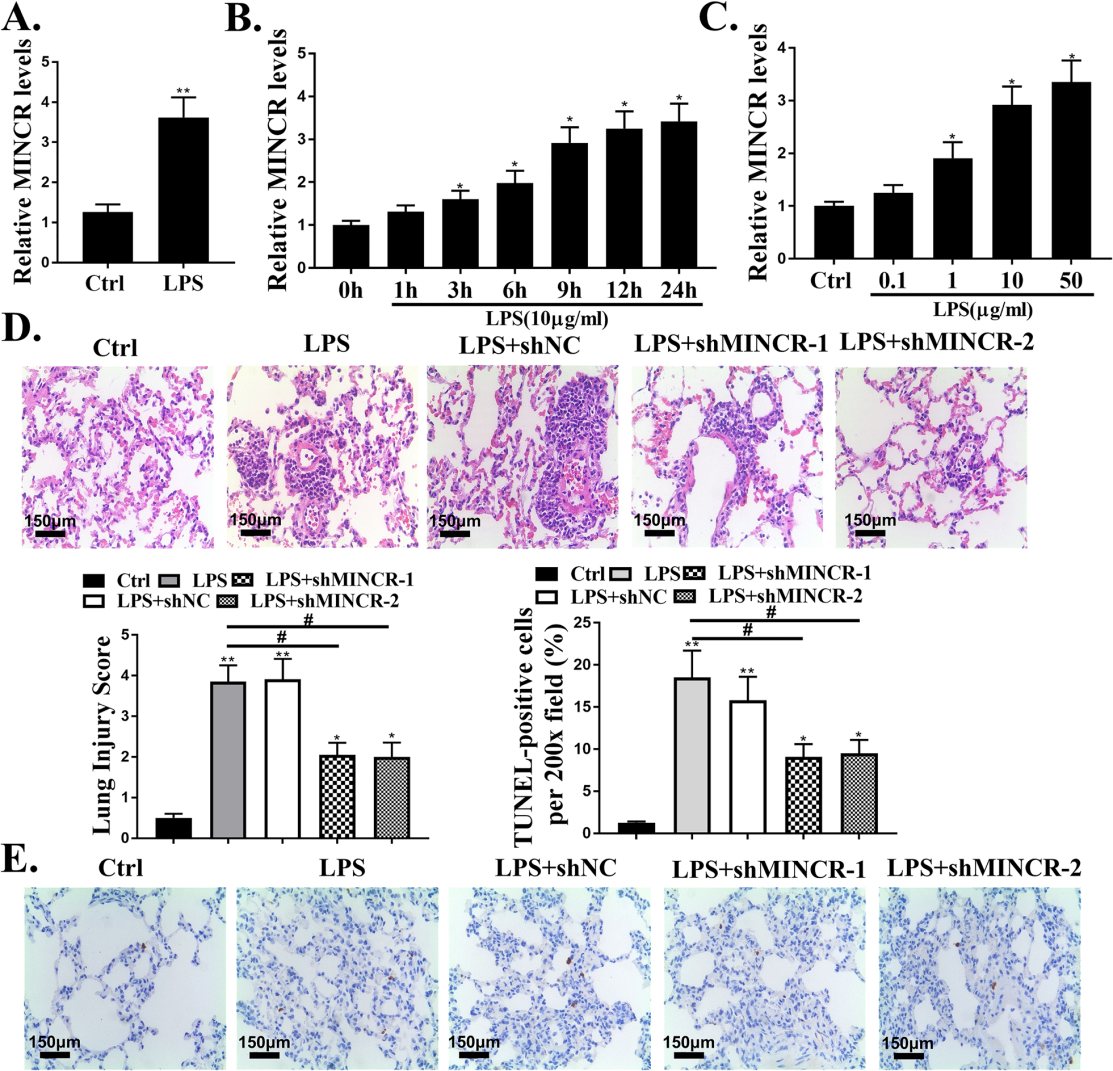
MINCR was upregulated in acute lung injury (ALI) mouse model and lipopolysaccharide (LPS)-injured alveolar epithelial cells. **A** the expression levels of MINCR in sepsis-induced ALI models. N = 10. **B** small airway epithelial cells (SAECs) were treated with different doses of LPS for 12 h, and then the expression level of MINCR was analyzed (N = 3). **C** after exposure to LPS for a specified period of time, the expression level of MINCR in SAECs was analyzed (N = 5). **D** hematoxylin and eosin staining (× 200) was used to observe the histological changes. Scale bar = 50 μm. **E** terminal deoxynucleotidyl transferase biotin-dUTP nick end labeling staining was used to observe the histological changes (×200). Scale bar = 50 μm. *p < 0.05, **p < 0.01, ***p < 0.001 vs. Ctrl group; ^#^*p* < 0.05 vs LPS. N = 10

Compared to the control group, the expression levels of MINCR in the lung tissue of LPS-injured mice were significantly increased (*P* < 0.05, Fig. 1A). SAECs were exposed to LPS to simulate ALI in vitro, and the expression levels of MINCR gradually increased with an increase in the duration of LPS and an increase in the concentration of the effect (*P* < 0.05, Fig. 1B and C). H&E staining results showed that the lung tissues with LPS injury showed thickened alveolar walls, pulmonary edema, and alveolar hemorrhage compared to the control group. ShMINCR significantly decreased LPS-induced lung tissue damage (Fig. 1C). Compared with the control group, the number of apoptotic cells in the lung tissues of LPS-injured mice was significantly increased (*P* < 0.05), indicating that the nuclear volume was reduced, the nuclear chromatin was concentrated and condensed, and apoptotic bodies were observed. ShMINCR significantly reduced the number of apoptotic cells in the lung tissues of mice (*P* < 0.05, Fig. 1D).

### Downregulated MINCR reduces neutrophilic inflammation in lungs of ARDS mouse model

**Fig. 2:**
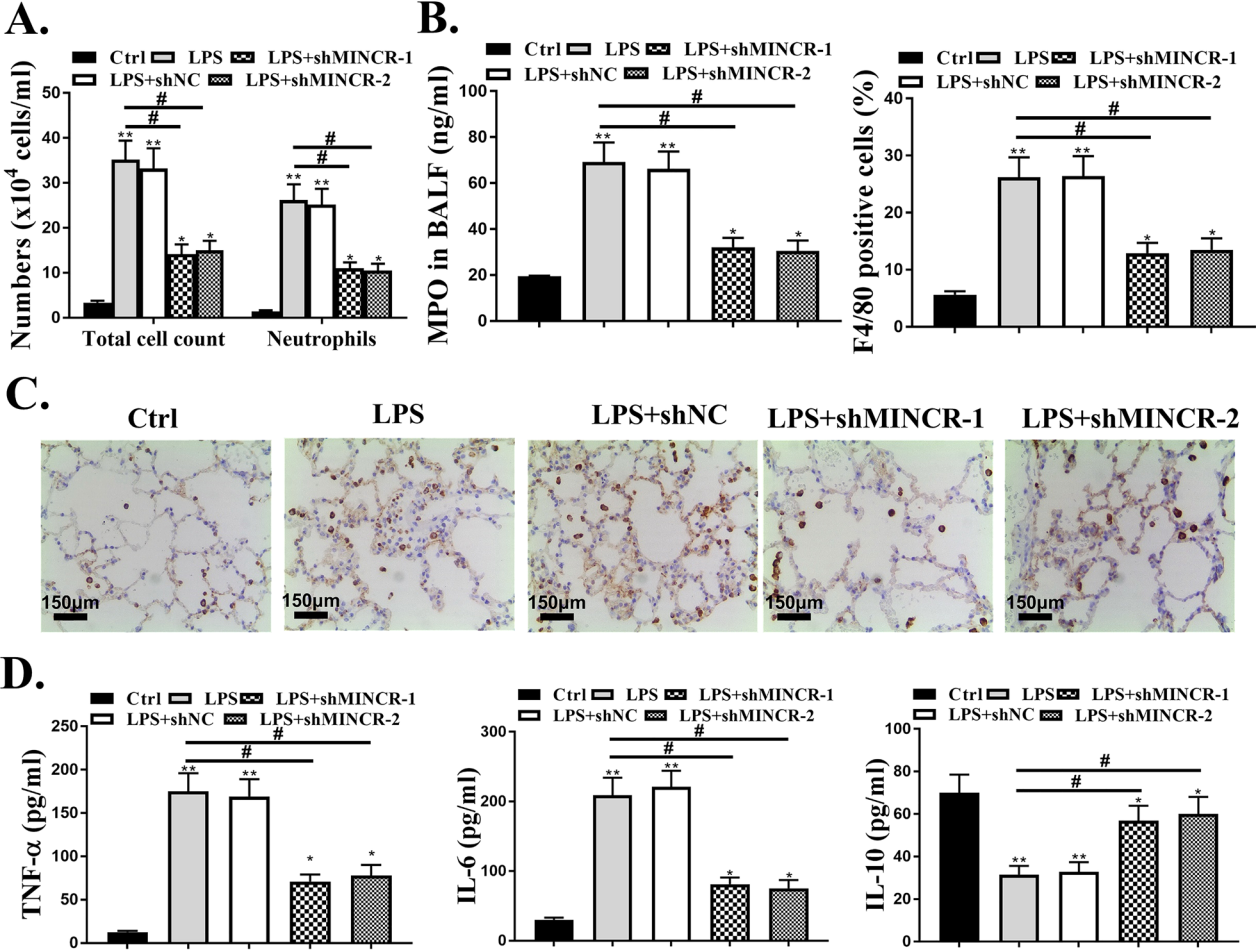
Effect of MINCR on neutrophil inflammation in lungs. **A** quantification of total cells and neutrophils in bronchoalveolar lavage fluid (BALF) of mice. **B** quantification of myeloperoxidase MPO activity in BALF of mice. **C** F4/80 IHC. Scale bar = 50 μm. **D** quantification of levels of TNF (tumor necrosis factor)-α, IL (interleukin)-6, and IL-10 in BALF of mice as examined by enzyme-linked immunosorbent assay. *p < 0.05, **p < 0.01, ***p < 0.001 vs. Ctrl group; ^#^*p* < 0.05 vs. LPS. N = 10

Thereafter, the effect of MINCR on pulmonary neutrophil inflammation was analyzed. Compared with the control group, the total cell number and the number of neutrophils in the BALF of the LPS group were significantly increased (*P* < 0.05). Post sh-MINCR treatment, the total number of cells and the number of neutrophils in the BALF were significantly decreased (*P* < 0.05, Fig. 2A). In addition, MPO activity, the number of F4/80 positive cells, and the expression levels of inflammatory factors (TNF-α and IL-6) in the LPS group were significantly increased (*P* < 0.05), while the expression levels of IL-10 were significantly reduced (*P* < 0.05). Post sh-MINCR treatment, the number of F4/80 positive cells and the expression levels of inflammatory factors (TNF-α and IL-6) were significantly reduced (*P* < 0.05). In contrast, the expression levels of IL-10 were significantly increased (*P* < 0.05, Fig. 2B–D).

### MiR-146b-5p directly regulated MINCR

**Fig. 3:**
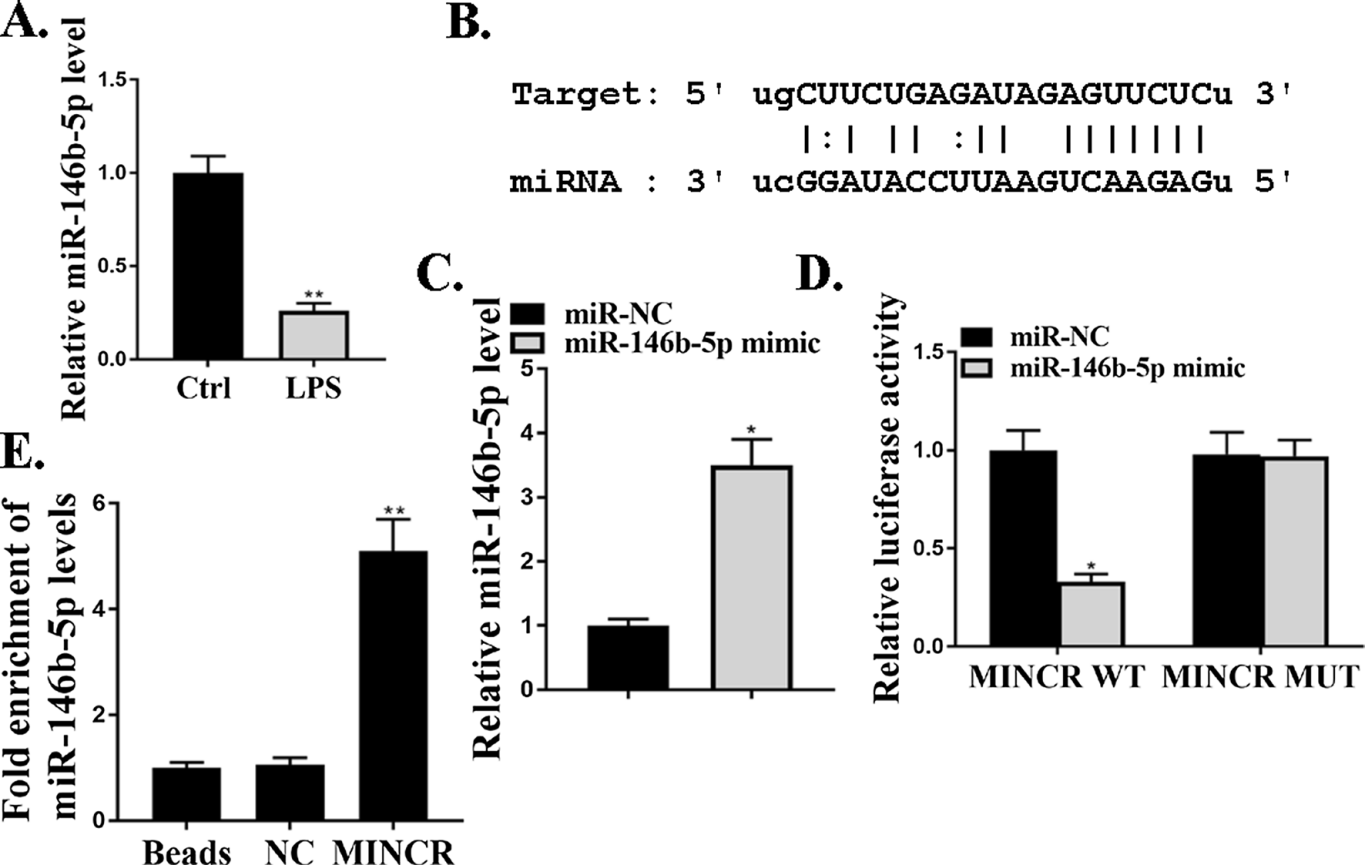
MiR-146b-5p directly regulated MINCR. **A** miR-146b-5p expression level in a model of acute lung injury (ALI) induced by lipopolysaccharide (LPS), N = 10. **B** MiR-146b-5p was predicted as a potential target of MINCR (**C**) miR-146b-5p expression level after mimic treatment. **D** analysis of luciferase activity in cells co-transfected with MINCR-wild type (WT) or MINCR-Mut vector. **E** immunoprecipitation test was used to determine the enrichment of MINCR and miR-146b-5p in the immunoprecipitation complex. Bars indicated standard deviation. *P < 0.05, **p < 0.01, N = 3

The mechanism by which MINCR regulated the progression of ALI was explored. Compared to the control group, the expression levels of miR-146b-5p were significantly reduced in the lung tissues of LPS-injured mice (*P* < 0.05, Fig. 3A). The online prediction tool Starbase v2.0 and miR-146b-5p was identified as a potential target of MINCR (Fig. 3B). As shown in Fig. 3C, compared to the miR-NC group, the expression levels of miR-146b-5p in the miR-146b-5p mimic group were significantly increased, indicating that the transfection was successful (*P* < 0.05). Luciferase activity was significantly reduced in cells co-transfected with miR-146b-5p and MINCR-WT (*P* < 0.05); however, the luciferase activity of MINCR-MUT did not change (Fig. 3D). In addition, MINCR and miR-146b-5p were preferentially enriched in miRNAPs containing Ago2 compared to anti-IgG immunoprecipitates (Fig. 3E). These results indicate that MINCR directly targets miR-146b-5p in ALI.

### MiR-146b-5p mediated the protective effects of sh-MINCR in LPS-induced ALI model

**Fig. 4:**
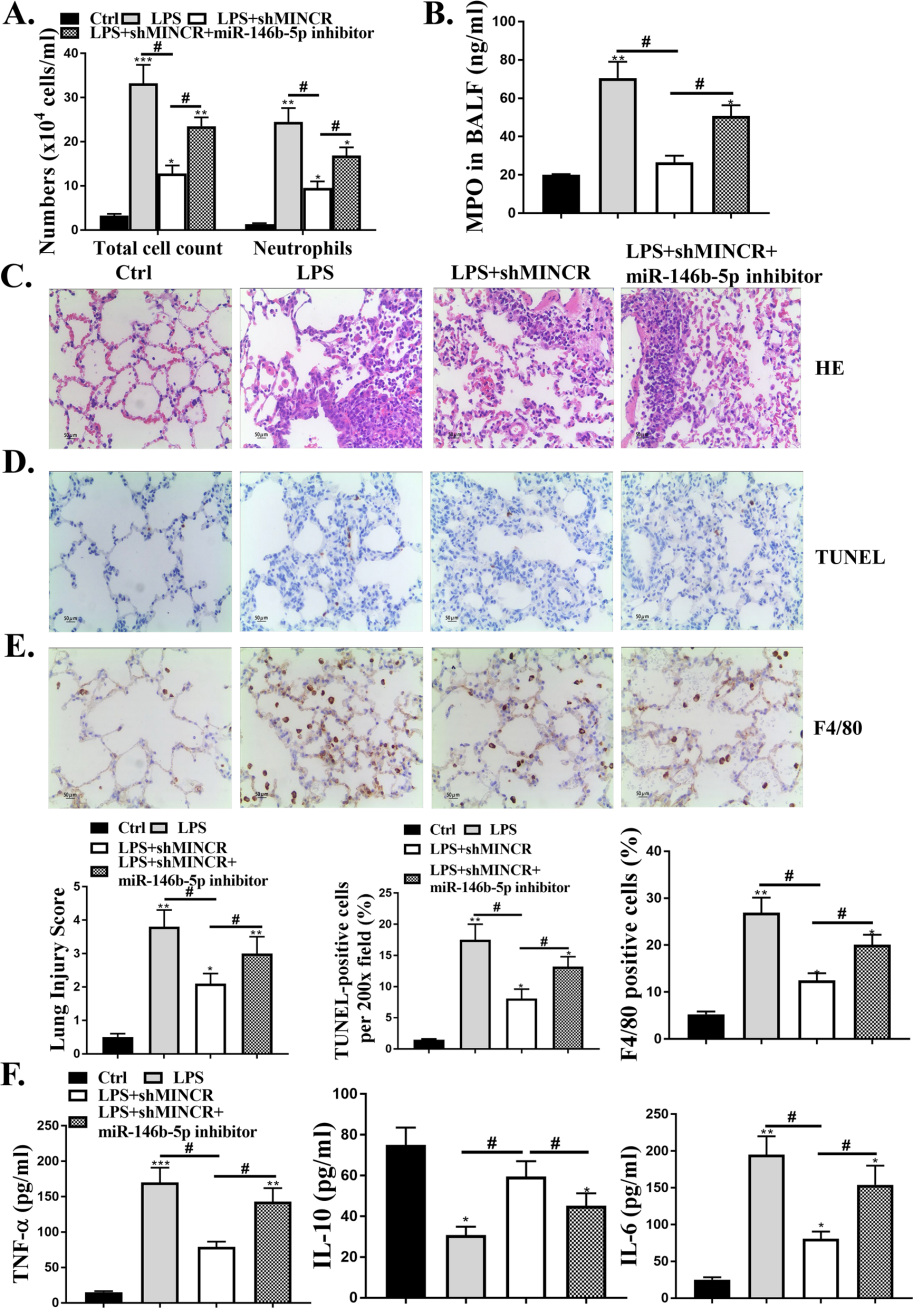
MiR-146b-5p-mediated sh-MINCR protected against lipopolysaccharide (LPS)-induced acute lung injury (ALI). **A** total and neutrophil counts in bronchoalveolar lavage fluid (BALF) mice. **B** mouse BALF myeloperoxidase (MPO) activity. **C** hematoxylin and eosin staining. Scale bar = 50 μm. **D** terminal deoxynucleotidyl transferase biotin-dUTP nick end labeling staining was used to detect apoptosis. Scale bar = 50 μm. **E** F4/80 IHC staining. Scale bar = 50 μm. **F** quantification of levels of TNF (tumor necrosis factor)-α, IL (interleukin)-6, and IL-10 were examined by enzyme-linked immunosorbent assay. *p < 0.05, **p < 0.01, ***p < 0.001 vs. Ctrl group; ^#^*p* < 0.05 vs. LPS. N = 10.

Furthermore, we explored whether MINCR affects lung neutrophil inflammation through miR-146b-5p. sh-MINCR significantly reduced the total amount of BALF and neutrophil count, and MPO activity was significantly reduced after LPS treatment (*P* < 0.05). Co-transfection of Sh-MINCR with miR-146b-5p inhibitor reversed the effects of sh-MINCR on total cell number, neutrophil count, and MPO activity (*P* < 0.05, Fig. 4A and B). H&E staining results showed that shMINCR significantly reduced LPS-induced lung tissue damage, whereas co-transfection of Sh-MINCR with miR-146b-5p inhibitor enhanced lung tissue damage (Fig. 4C). shMINCR significantly reduced the number of apoptotic cells, whereas co-transfection of sh-MINCR with miR-146b-5p inhibitor increased the number of apoptotic cells in lung tissues (*P* < 0.05, Fig. 4D). As shown in Fig. 4E and F, sh-MINCR significantly reduced the number of F4/80 positive cells and the expression levels of inflammatory factors (TNF-α and IL-6) in vivo after LPS treatment (*P* < 0.05); the expression levels of IL-10 were significantly increased (*P* < 0.05), whereas co-transfection of sh-MINCR with miR-146b-5p inhibitor reversed the effects of sh-MINCR on MPO activity, number of F4/80 positive cells, and expression levels of inflammatory factors (*P* < 0.05).

### MiR-146b-5p mediated the protective effects of sh-MINCR on LPS-induced cell apoptosis and inflammation in SAECs

**Fig. 5:**
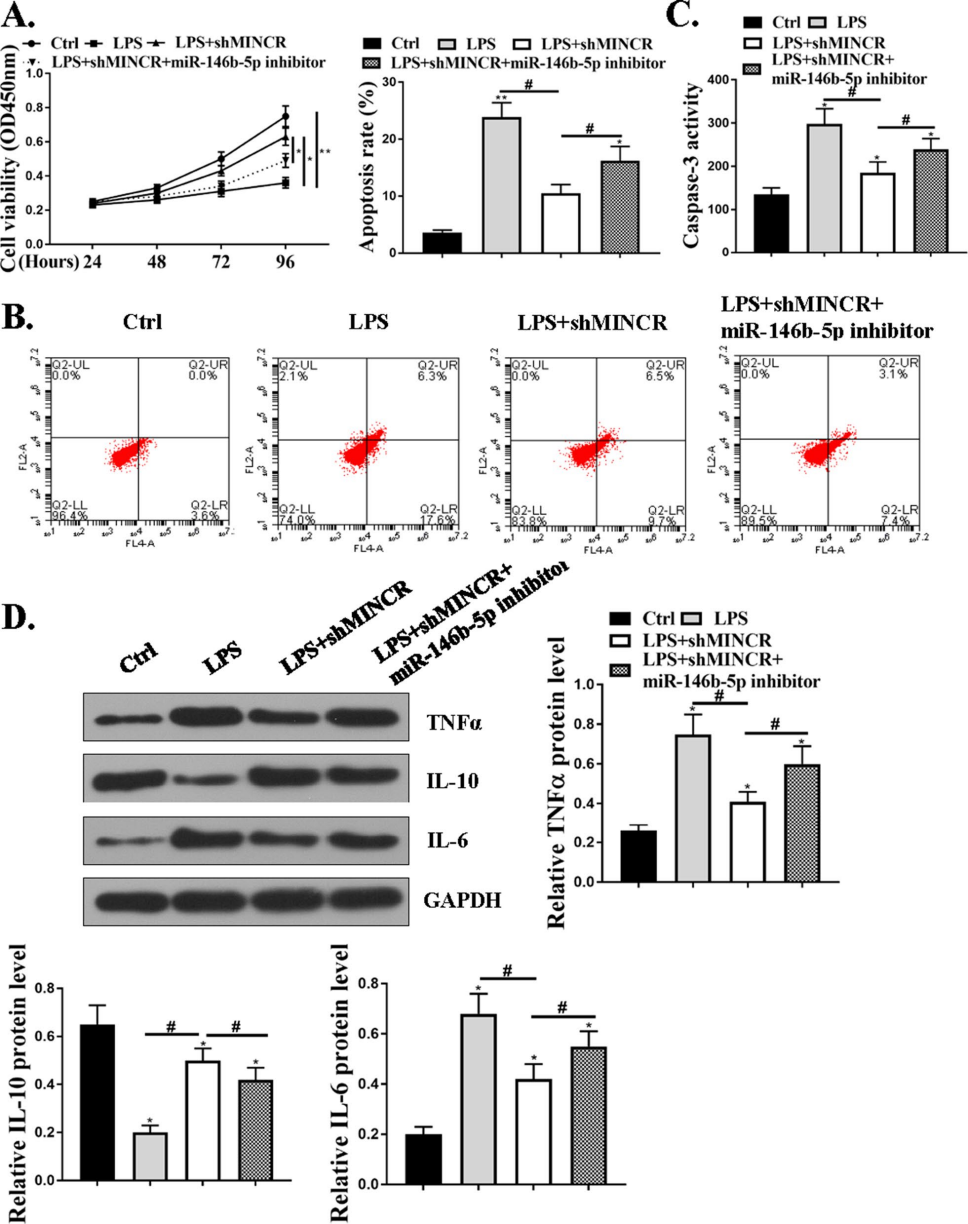
MiR-146b-5p mediated the protective effect of sh-MINCR on lipopolysaccharide (LPS)-induced apoptosis and inflammation of small airway epithelial cells (SAECs). **A** cell viability was determined by CCK8. **B** apoptosis was detected by flow cytometry. **C** Caspase-3 activity assessment of different groups. **D** TNF (tumor necrosis factor)-α, IL (interleukin)-6 and IL-10 protein expression levels in SAECs was detected by Western blotting assay. *P < 0.05, **p < 0.01 vs. Ctrl group; ^#^*p* < 0.05 vs. LPS group. N = 5

Compared with the control group, the SAECs in the LPS group had significantly reduced viability and increased death rate. The viability of SAECs was significantly increased and the apoptosis rate was significantly reduced after treatment with sh-MINCR, whereas co-transfection of sh-MINCR with miR-146b-5p inhibitor reversed the effect of sh-MINCR on the viability and apoptosis of SAECs (*P* < 0.05, Fig. 5A and B). Caspase-3 activity was significantly elevated in the LPS group compared to the control group (Fig. 5C). In addition, compared with the control group, the expression levels of TNF-α and IL-6 protein in the LPS group were significantly increased and the expression levels of IL-10 protein were significantly reduced. After treatment with sh-MINCR, the expression levels of TNF-α and IL-6 proteins were significantly reduced and the expression levels of IL-10 protein were significantly increased, while co-transfection of sh-MINCR with miR-146b-5p inhibitor reversed the effect of shMINCR on the expression of cellular inflammatory factors (*P* < 0.05, Fig. 5D).

### MiR-146b-5p directly targeted the 3′-UTR of TRAF6 mRNA

**Fig. 6:**
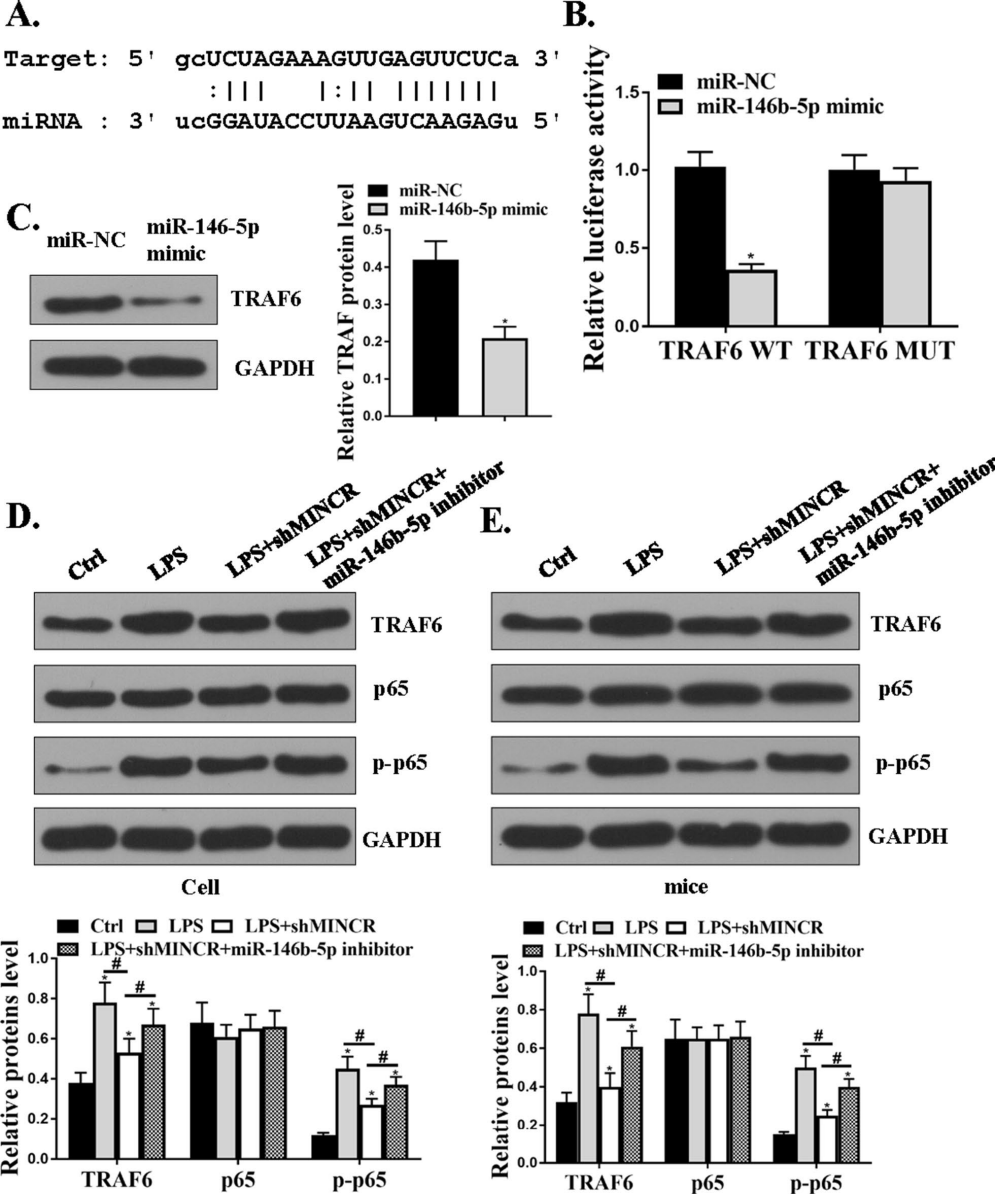
MINCRT affected the progression of acute lung injury (ALI) through the miR-146b-5p/TRAF6 axis. **A** TRAF6 was predicted to be a target gene of miR-146b-5p. **B** analysis of luciferase activity in TRF6-wild type (WT) or TRAF6-Mut vector co-transfected cells. **C** protein expression levels of TRAF6 in small airway epithelial cells (SAECs). **D** protein expression levels of TRAF6, P65, and p-P65 in SAECs was detected by Western blotting assay. **E** protein levels of TRAF6, p65, and p-P65 in lung tissue treated with lipopolysaccharide (LPS) was detected by Western blotting assay. *P < 0.05, N = 5

The online prediction tool, Starbase v2.0, was used and TRAF6 was identified as a potential target for miR-146b-5p (Fig. 6A). The luciferase activity of miR-146b-5p mimic and TRAF6-WT co-transfected cells was significantly reduced (*P* < 0.05); however, the luciferase activity of TRAF6-MUT did not change (Fig. 6B). Compared to the miR-NC group, the expression levels of TRAF6 protein in the miR-146b-5p group were significantly reduced (*P* < 0.05, Fig. 6C). As shown in Fig. 6D and E, compared to the control group, LPS significantly increased the expression levels of TRAF6 and p-P65 in the lung tissues of SAECs (*P* < 0.05), and sh-MINCR significantly reduced the expression levels of TRAF6 and p-P65 in the lung tissues of LPS-induced SAECs, whereas co-transfection of sh-MINCR with miR-146b-5p inhibitor reversed the effect of sh-MINCR on the expression levels of TRAF6 and p-P65. These results indicated that MINCR increased the expression levels of TRAF6 and p-P65 by regulating miR-146b-5p.

## Discussion

ALI/ARDS is an acute and critical illness of the respiratory system (Fu and Thangavel 2019). The imbalance of inflammatory response, increase in alveolar permeability, and abnormal activation of the coagulation pathway caused by the destruction of epithelial cells and vascular endothelial cells are the main characteristics of ALI/ARDS pathogenesis (Tong 2018). The causes of ALI/ARDS are diverse and their pathogenesis is highly complicated. Therefore, an in-depth understanding of the occurrence, development, and regulatory mechanisms of ALI is essential. Due to the limitation of studying the pathological changes in the lungs in vivo, animal models are the best choice to study ALI (Wang et al. 2018). Therefore, LPS was used to induce ALI in this study. After LPS injection, there was a significant lung injury in the mice, indicating that ALI was successfully induced.

The biological functions of lncRNAs vary. They regulate the expression of specific genes in the process of recruitment, post-transcription modification, and epigenetics of transcription (Yuchen et al. 2020). Studies have shown that lncRNAs regulate gene expression at different levels, such as the transcriptional level of ALI, and participate in the development, infiltration, and transfer of ALI. For example, lncRNA MALAT1 is negatively regulated by BMI-111. Upregulation of BMI-111 can reduce the hypertonicity of endothelial cells by downregulating lncRNA MALAT1, reducing LPS-induced lung injury (Li et al. 2018b). In addition, an experimental model of pulmonary endothelial inflammation and barrier dysfunction was established by LPS stimulation of HPMECs. lncRNAs play a key role in LPS-induced pulmonary endothelial cell inflammation and barrier dysfunction (Li et al. 2018c). MINCR is a recently discovered lncRNA. In this study, MINCR was found to be upregulated in the lung tissues of LPS-injured mice and SAECs. ShMINCR reduced LPS-induced lung tissue damage and the number of apoptotic cells in the lung tissue of mice. Sh-MINCR significantly reduced the total cell number, neutrophil number, MPO activity, F4/80 positive cell number, and the expression levels of inflammatory factors (TNF-α and IL-6) in LPS-induced BALF, and increased the expression levels of IL-10. After sh-MINCR treatment, the viability of SAECs increased and the apoptosis rate was reduced. It was speculated that MINCR plays a key role in the development of ALI inflammation caused by LPS, and that the knockout of PVT1 had a protective effect on LPS-induced ALI inflammation and cell viability.

Studies have shown that miRNAs are involved in regulating physiological processes, such as insulin secretion, tumor formation, bacterial infection, and viral infection (Li et al. 2019). It was reported that miRNAs play a role in lung diseases such as lung growth and development, pneumonia, lung cancer, and pulmonary fibrosis (Hou et al. 2018). For example, the expression levels of miR-155 in the lung tissues of WT mice were significantly increased compared to those in miR-155 inhibitor mice. It can inhibit its expression and regulate the pulmonary inflammatory response by binding to the downstream target gene cytokine signal suppressor (Feng et al. 2016). As a member of the miRNA, miR-146b-5p plays different roles in different diseases (Xu et al. 2018). In this study, miR-146b-5p was identified as a target gene for MINCR. The expression levels of miR-146b-5p were significantly reduced in LPS-induced ALI lung tissues. Co-transfection of sh-MINCR with miR-146b-5p inhibitor increased the number of lung tissue damage and apoptosis. Simultaneously, co-transfection of sh-MINCR with miR-146b-5p inhibitor reversed the effects of sh-MINCR on the total cell number, neutrophil number, F4/80 positive cell number, the expression levels of inflammatory factors (TNF-α, IL-6, and IL-10), and the activity and apoptosis of SAECs. These results indicate that MINCR regulates ALI injury through miR-146b-5p.

The tumor necrosis factor receptor related factor family (TRAF) is a conserved linker protein (Zhu et al. 2018). TRAF6 is closely related to ALI caused by sepsis, and overexpression of TRAF6 increases the expression levels of TNF-α and IL-6 (Wu et al. 2019b). Both p38 MAPK and NF-κB are involved in regulating the uncontrolled inflammatory response in AM (Tian et al. 2019). Activated P38 MAPK promotes the expression of AM and synthesizes and releases inflammatory transmitters and arachidonic acid metabolites, which are involved in the inflammatory process (Zhang et al. 2019). When AM is stimulated by inflammation, NF-κB induces AM to secrete a variety of inflammatory transmitters and aggravates lung injury (Wu et al. 2019a). Studies have shown that TRAF6 can activate the NF-κB signaling pathway (Ji et al. 2018). This study found that TRAF6 is a target gene of miR-146b-5p. Sh-MINCR reduced the expression levels of TRAF6 and p-P65 in LPS-induced SAECs and lung tissues of mice. Co-transfection of sh-MINCR with miR-146b-5p inhibitor reversed the effect of sh-MINCR on the expression of TRAF6 and p-P65. These data demonstrated that MINCR enhanced the expression levels of TRAF6 and p-P65 by regulating miR-146b-5p.

## Conclusion

MINCR regulates ALI damage by upregulating the expression of TRAF6 and p-P65 through miR-146b-5p. Thus, it is a potential target for the treatment of ALI.

## Data Availability

The data that support the findings of this study are available upon request from the corresponding author. The data are not publicly available because they contain information that can compromise the privacy of research participants.

## Abbreviations

ALI: Acute lung injury
ARDS: Acute respiratory distress syndrome
BALF: Bronchoalveolar lavage fluid
ELISA: Enzyme-linked immunosorbent assay
FBS: Fetal bovine serum
H&E: Hematoxylin and eosin
IL: Interleukin
lncRNA: Long non-coding RNA
LPS: Lipopolysaccharide
WT: Wild type
MPO: Myeloperoxidase
PMNs: Polymorphonuclear leucocytes
qRT-PCR: Quantitative real-time polymerase chain reaction
SAECs: Small airway epithelial cells
TNF: Tumor necrosis factor
TNAF: Tumor necrosis factor receptor related factor family
TU: Transducing units
TUNEL: Terminal deoxynucleotidyl transferase biotin-dUTP nick end labeling
